# Correction: Peroxisome proliferator–activated receptor (PPAR)α expression in T cells mediates gender differences in development of T cell–mediated autoimmunity

**DOI:** 10.1084/jem.2006183905142018c

**Published:** 2018-06-04

**Authors:** Shannon E. Dunn, Shalina S. Ousman, Raymond A. Sobel, Luis Zuniga, Sergio E. Baranzini, Sawsan Youssef, Andrea Crowell, John Loh, Jorge Oksenberg, Lawrence Steinman

Vol. 204, No. 2, February 19, 2007. 10.1084/jem.20061839.

After publication, the authors were made aware of incorrect primer sequences listed for PPARα in the Mice section of Materials and methods.

The correct primer sequences used to amplify PPARα cDNAs are as follows:

Sense: 5**′**-GAGAAGTTGCAGGAGGGGATTGTG-3**′**

Antisense: 5**′**-CCCATTTCGGTAGCAGGTAGTCTT-3**′**

